# Attitudes, Behaviors, and Perceived Barriers Toward Conducting Research Among Preventive Medicine Residents in Saudi Arabia: A National Cross-Sectional Study

**DOI:** 10.7759/cureus.88303

**Published:** 2025-07-19

**Authors:** Amal A Alghamdi, Abeer A Subke

**Affiliations:** 1 Preventive Medicine Postgraduate Program, Saudi Ministry of Health, Jeddah, SAU

**Keywords:** kingdom of saudi arabia (ksa), medical residency, postgraduate medical education, preventive medicine board, willing to research

## Abstract

Objectives

This study aimed to evaluate the attitudes, behaviors, and practices of preventive medicine residents in Saudi Arabia in 2024, and identify the perceived barriers and challenges they face.

Methods

This analytical cross-sectional study was conducted among preventive medicine residents registered in the Saudi Commission for Health Specialties (SCFHS) at all levels (years one to four) between January and April 2024. Data were collected via a self-administered online survey questionnaire covering sociodemographic characteristics, attitudes, and practices towards research, motivation to conduct research, and perceived barriers. Descriptive statistics, chi-square tests, and Fisher's exact tests were used for data analysis.

Results

A total of 182 residents participated in this study. The majority were aged 25-30 years (61.0%) and male (53.8%). Most participants were in their fourth year of residency (29.1%) and married (57.7%). Most articles had previous publications in journals (80.2%). Significant associations were found between the demographic characteristics and publication. Residents expressed their willingness to participate in the research workshops. The primary motives were having a positive impact on CV, improving research skills, and becoming published. The main perceived barriers included a lack of time, inadequate research facilities, and poor database accessibility.

Conclusion

These findings can inform targeted interventions to foster research culture and address specific challenges, ultimately improving healthcare outcomes and shaping future public health policies.

## Introduction

Medical research is the cornerstone of developing and enhancing knowledge in the medical field [1]. It provides a better understanding of diseases, supports the development of effective treatments, and improves healthcare outcomes [1-3]. It constitutes the foundation of evidence-based medicine, enabling healthcare professionals to make informed decisions and provide the best possible care to patients [2-4]. Consequently, engaging in research activities is of the utmost importance to postgraduate doctors [5]. Undertaking research enables them to dive deeper into their chosen field of specialization and expand their knowledge beyond the boundaries of clinical practice [5].

Previous studies have comprehensively evaluated the involvement of medical postgraduate physicians in research projects [6]. Overall, medical postgraduate doctors consistently display a favorable attitude toward actively participating in research projects [6,7]. This emphasizes the importance of medical research in improving patient care, enabling professional enhancement, and changing health policy [2-10]. However, the enrollment rate of residents in research during their residency training reflects the opposite [7]. In India, the percentage of postgraduate doctors who have conducted research ranges from 4% to 31% [8,9]. In Saudi Arabia, the rate fluctuates by specialty [2]. It ranges from 16% among postgraduate doctors in family medicine to 0.4% among those in cardiac surgery [2]. Specialties with positive relative contributions to research productivity include ophthalmology, miscellaneous medicine, and pediatrics [11]. However, despite ranking eighth in total research contributions, public health specialties have not had a positive relative impact on research productivity in Saudi Arabia in the past two decades [11].

Doctors across diverse medical specialties encounter several obstacles that prevent them from participating in academic pursuits [2]. A study undertaken in India demonstrated that a lack of time and a heavy workload, primarily attributed to the extensive postgraduate curriculum (59.5%), posed a significant challenge to research participation [9]. Additionally, inadequate research facilities (25.8%) and the absence of a dedicated research curriculum (25%) emerged as identified hurdles [9]. In Saudi Arabia, difficulty in publishing (37%) and a lack of statistical support (36%) were the chief obstacles encountered by physicians [12]. Furthermore, it is worth noting that approximately half of the residents in a study expressed unwillingness to conduct research [12]. The limited participation of postgraduate doctors in research activities stems from several factors [2-4,12]. These include a lack of training in research methodologies and skills, limited time due to their demanding work, inadequate supervision and mentorship, restricted infrastructure and resources, and a lack of interest in or motivation for research [2-4,6,7,12,13]. Studies conducted among postgraduate doctors in Riyadh revealed that the biggest obstacle to undertaking research was a lack of research training, followed by a lack of time [14]. Addressing these barriers through targeted training programs, structured time allocation, enhanced supervision, improved infrastructure, and financial support can promote greater resident engagement in research [13,14].

The Saudi Vision 2030 Program prioritizes research and development to achieve international standards in higher education [15]. In line with this vision, the Saudi Postgraduate Preventive Medicine Program recognizes the significance of research and integrates it as a cornerstone component of its curriculum [16]. Preventive medicine is a medical specialty that focuses on population health and clinical practice, enabling physicians to address threats to public health and work toward promoting well-being and preventing diseases, disabilities, and mortality [16]. The program is supervised and evaluated by the Saudi Commission for Health Specialties (SCFHS) [16]. The Saudi Postgraduate Preventive Medicine Program achieves its goals through dedicated academic courses, time allocated for research activities, and the requirement for postgraduate doctors to undertake at least two research projects [16]. Optimistically, this will inspire preventive medicine professionals and equip them with the skills to analyze existing knowledge and advance the field of disease prevention [16].

To the best of our knowledge, this study represents the first national survey investigating the attitudes, behaviors, and perceived obstacles related to research engagement among postgraduate doctors in preventive medicine in Saudi Arabia. Given the significant gap in the literature and the limited involvement of these residents in research activities, This study aimed to: (1) assess attitudes, behaviors, and practices toward research among preventive medicine residents in Saudi Arabia, (2) identify perceived barriers to conducting research, and (3) explore how demographic factors relate to research engagement and activities. By addressing this critical gap, we sought to provide insights into the dynamics of research involvement among preventive medicine residents in Saudi Arabia.

## Materials and methods

Study design and population

An analytical cross-sectional study was performed among preventive medicine residents registered with SCFHS at all levels (years one to four) between January and April 2024. Individuals from other specialties and those who refused to participate were excluded from this study.

Sample size and sampling technique

The Raosoft sample size calculator (Raosoft, Inc., Seattle, Washington) was used to determine the minimum sample size required for the study. Assuming that 50% of the participants possessed good knowledge, with a 5% margin of error and a 95% confidence interval, the estimated sample size of preventive medicine residents was 183. This sample size was selected to ensure sufficient statistical power to detect meaningful differences and enhance the reliability of our findings, while also considering the total number of registered preventive medicine residents in Saudi Arabia. All eligible residents were invited to participate via official email channels. A convenience sampling method was employed, relying on voluntary responses to the online invitation, which may limit the generalizability of the findings.

Data were collected using a Google survey link containing the survey questionnaire. The survey link was distributed among the participants through official email. The invitation letter for the survey began with a detailed explanation of the study’s objectives and aims. It also clarified that completing this questionnaire did not entail any financial benefit or degree. Participation was voluntary and informed consent (electronic consent) was obtained from all participants. Additionally, to maximize participation and address any potential technical issues, the email invitation was resent after two weeks to ensure that all participants had the opportunity to receive and respond to the questionnaire.

Data sources and collection

Attitudes, behaviors, and perceived barriers related to conducting research among preventive medicine residents were measured using a self-administered questionnaire developed by Soubhanneyaz et al. with permission [12]. The internal consistency of the total attitude score yielded a Cronbach's alpha value of 0.87. The questionnaire comprised four sections. The first part included sociodemographic characteristics, such as age, gender, academic year, and marital status. The second part consisted of nine (yes/no) questions that examined attitudes and practices toward research, including previous experiences in publication and presentation. Publication was defined as authorship in a peer-reviewed journal, such as case reports, original articles, or review papers, or as the presentation of research findings at a scientific conference. The third part consisted of five items on a five-point Likert scale, ranging from 1 (strongly disagree) to 5 (strongly agree), which investigated the motivation of preventive medicine residents to conduct research. The last part consisted of 21 items, using a five-point Likert scale that ranged from 1 (strongly disagree) to 5 (strongly agree), to evaluate the barriers that may have deterred preventive medicine residents from engaging in research projects.

Statistical analysis

The Statistical Package for the Social Sciences (SPSS® version 26.0, IBM, Armonk, NY) was used for statistical analyses. Descriptive statistics summarized participants' demographics, motives, perceived barriers, attitudes, practices, and research contributions. Categorical variables were expressed as frequency and percentage. The data were tested for normal distribution using the Kolmogorov-Smirnov test. Moreover, the associations between demographic characteristics and publication status, as well as residents' willingness to conduct research, were analyzed using Pearson's chi-square test and Fisher's exact test, where appropriate. Statistical significance was set at p < 0.05. Incomplete responses were excluded from the final analysis. No imputation was performed for missing data.

Ethics

The study protocol was reviewed and approved by the Research Ethics Committee of the Ministry of Health, Jeddah, Saudi Arabia (reference number A01812). Informed consent was obtained from all participants. The anonymity and confidentiality of the information were ensured.

## Results

Demographic characteristics and their association with having a publication

In this study, we analyzed data from 182 residents (response rate 50.4%). The majority were aged 25-30 years (61.0%) and male (53.8%). Most participants were in their fourth year of residency (29.1%) and married (57.7%). Regarding the program city, the highest proportions of residents were from Jeddah (23.6%) and Riyadh (17.6%), as shown in Table 1. Most residents (80.2%) published articles in journals. The association between demographic characteristics and journal publication revealed significant differences based on age (p = 0.022), residence year (p = 0.008), and program city (p = 0.002). Specifically, residents aged 25 to 30 years (85.6%) and ≥35 years (85.6%) had significantly higher publication rates than those aged 30 to 35 years (67.3%). Additionally, R1 residents were more likely to have publications (92.7%; Table 1).

**Table 1:** Demographic characteristics and their association with having a publication. *Fisher's exact test; Pearson's Chi-squared test. **Significant level at p < 0.05.

Characteristic	Description	Having any publication in a journal		Statistic* and P-value**
		No, N = 36	Yes, N = 146	
Age				
25 to <30	111 (61.0%)	16 (14.4%)	95 (85.6%)	χ² = 7.668, p = 0.022
30 to <35	52 (28.6%)	17 (32.7%)	35 (67.3%)	
35 or more	19 (10.4%)	3 (15.8%)	16 (84.2%)	
Gender				
Female	84 (46.2%)	14 (16.7%)	70 (83.3%)	χ² = 0.953, p = 0.329
Male	98 (53.8%)	22 (22.4%)	76 (77.6%)	
Residency year				
R1	41 (22.5%)	3 (7.3%)	38 (92.7%)	χ² = 11.783, p = 0.008
R2	38 (20.9%)	5 (13.2%)	33 (86.8%)	
R3	50 (27.5%)	10 (20.0%)	40 (80.0%)	
R4	53 (29.1%)	18 (34.0%)	35 (66.0%)	
Marital status				
Single	77 (42.3%)	12 (15.6%)	65 (84.4%)	χ² = 1.481, p = 0.224
Married	105 (57.7%)	24 (22.9%)	81 (77.1%)	
Program city				
Abha	13 (7.1%)	8 (61.5%)	5 (38.5%)	χ² = 27.887, p = 0.002
Albaha	1 (0.5%)	0 (0.0%)	1 (100.0%)	
Alhasa	24 (13.2%)	5 (20.8%)	19 (79.2%)	
Jazan	14 (7.7%)	4 (28.6%)	10 (71.4%)	
Jeddah	43 (23.6%)	7 (16.3%)	36 (83.7%)	
Khamis Mushait	4 (2.2%)	2 (50.0%)	2 (50.0%)	
Madina	14 (7.7%)	1 (7.1%)	13 (92.9%)	
Makkah	5 (2.7%)	0 (0.0%)	5 (100.0%)	
Riyadh	32 (17.6%)	1 (3.1%)	31 (96.9%)	
Tabuk	17 (9.3%)	3 (17.6%)	14 (82.4%)	
Taif	15 (8.2%)	5 (33.3%)	10 (66.7%)	

Participants' attitudes, practices, research contributions, and project types

The majority of participants expressed willingness to participate in workshops on conducting community-related research (91.2%), research methodology (86.3%), and conducting clinical research (75.8%). Most participants had previously participated in a study (96.2%). Their contributions predominantly included data collection (75.8%) and writing the proposal (74.7%). The most common research design was cross-sectional (80.8%; Table 2).

**Table 2:** Participants' attitudes, practices, research contributions, and project types.

Characteristic	Description
Attitudes and practices of research	n (%)
Willingness to participate in a workshop on research methodology	157 (86.3%)
Willingness to conduct clinical-related research	138 (75.8%)
Willingness to conduct community-related research	166 (91.2%)
Previous participation in research	175 (96.2%)
Regular reading of journals	47 (25.8%)
Presentation of a poster or research paper at a conference	67 (36.8%)
Contribution in research	n (%)
Literature review	125 (68.7%)
Proposal writing	136 (74.7%)
Data collection	138 (75.8%)
Data entry	75 (41.2%)
Data analysis	53 (29.1%)
Writing the manuscript	92 (50.5%)
Submission of the manuscript to the journal	56 (30.8%)
I haven't participated in a research project	7 (3.8%)
Research project types	n (%)
Case report	22 (12.1%)
Case series	5 (2.7%)
Cross-section	147 (80.8%)
Case-control	16 (8.8%)
Clinical trial	4 (2.2%)
Review article	61 (33.5%)
I haven't participated in a research project	7 (3.8%)

Differences in participants' willingness to conduct research

Significant differences were found in willingness to conduct clinical research based on age (p = 0.048; Table 3), with significantly lower rates of willingness expressed by those aged 30 to 35 years (67.3%) than other age groups (76.6% for 25 to 30 years and 94.7% for 35 years or more). No significant differences were observed in willingness to participate in workshops on research methodology or conduct community-related research based on other demographic variables (Tables 3, 4).

**Table 3:** Differences in participants' willingness to do research. *Significant level at p < 0.05. **Fisher's exact test; Pearson's Chi-squared test.

Characteristic	Willingness to conduct clinical-related research		P-value* and test statistics**	Willingness to conduct community-related research		P-value* and test statistics**
	No, N = 44	Yes, N = 138		No, N = 16	Yes, N = 166	
Age						
25 to <30	26 (23.4%)	85 (76.6%)	p = 0.048, χ² = 5.991	10 (9.0%)	101 (91.0%)	p = 0.415, χ² = 1.759
30 to <35	17 (32.7%)	35 (67.3%)		3 (5.8%)	49 (94.2%)	
35 or more	1 (5.3%)	18 (94.7%)		3 (15.8%)	16 (84.2%)	
Gender						
Female	18 (21.4%)	66 (78.6%)	p = 0.423, χ² = 0.642	10 (11.9%)	74 (88.1%)	p = 0.170, χ² = 1.886
Male	26 (26.5%)	72 (73.5%)		6 (6.1%)	92 (93.9%)	
Residency year						
R1	5 (12.2%)	36 (87.8%)	p = 0.234, χ² = 4.265	3 (7.3%)	38 (92.7%)	p = 0.806, χ² = 1.215
R2	11 (28.9%)	27 (71.1%)		2 (5.3%)	36 (94.7%)	
R3	13 (26.0%)	37 (74.0%)		5 (10.0%)	45 (90.0%)	
R4	15 (28.3%)	38 (71.7%)		6 (11.3%)	47 (88.7%)	
Marital status						
Single	20 (26.0%)	57 (74.0%)	p = 0.628, χ² = 0.235	8 (10.4%)	69 (89.6%)	p = 0.514, χ² = 0.425
Married	24 (22.9%)	81 (77.1%)		8 (7.6%)	97 (92.4%)	
Program city						
Abha	5 (38.5%)	8 (61.5%)	p = 0.237, χ² = 12.775	1 (7.7%)	12 (92.3%)	p = 0.173, χ² = 13.999
Albaha	0 (0.0%)	1 (100.0%)		0 (0.0%)	1 (100.0%)	
Alhasa	6 (25.0%)	18 (75.0%)		4 (16.7%)	20 (83.3%)	
Jazan	3 (21.4%)	11 (78.6%)		0 (0.0%)	14 (100.0%)	
Jeddah	6 (14.0%)	37 (86.0%)		2 (4.7%)	41 (95.3%)	
Khamis Mushait	1 (25.0%)	3 (75.0%)		1 (25.0%)	3 (75.0%)	
Madina	3 (21.4%)	11 (78.6%)		2 (14.3%)	12 (85.7%)	
Makkah	0 (0.0%)	5 (100.0%)		0 (0.0%)	5 (100.0%)	
Riyadh	13 (40.6%)	19 (59.4%)		1 (3.1%)	31 (96.9%)	
Tabuk	2 (11.8%)	15 (88.2%)		1 (5.9%)	16 (94.1%)	
Taif	5 (33.3%)	10 (66.7%)		4 (26.7%)	11 (73.3%)	

**Table 4:** Differences in participants' willingness to participate in a workshop. *Fisher's exact test; Pearson's Chi-squared test. **Significant level at p < 0.05.

Characteristic	Willingness to participate in a workshop on research methodology		Statistic* and P-value**
	No, N = 25	Yes, N = 157	
Age			
25 to <30	15 (13.5%)	96 (86.5%)	χ² = 1.716, p = 0.424
30 to <35	9 (17.3%)	43 (82.7%)	
35 or more	1 (5.3%)	18 (94.7%)	
Gender			
Female	15 (17.9%)	69 (82.1%)	χ² = 2.236, p = 0.135
Male	10 (10.2%)	88 (89.8%)	
Residency year			
R1	6 (14.6%)	35 (85.4%)	χ² = 1.099, p = 0.777
R2	5 (13.2%)	33 (86.8%)	
R3	5 (10.0%)	45 (90.0%)	
R4	9 (17.0%)	44 (83.0%)	
Marital status			
Single	9 (11.7%)	68 (88.3%)	χ² = 0.472, p = 0.492
Married	16 (15.2%)	89 (84.8%)	
Program city			
Abha	0 (0.0%)	13 (100.0%)	χ² = 9.776, p = 0.460
Albaha	0 (0.0%)	1 (100.0%)	
Alhasa	4 (16.7%)	20 (83.3%)	
Jazan	0 (0.0%)	14 (100.0%)	
Jeddah	7 (16.3%)	36 (83.7%)	
Khamis Mushait	1 (25.0%)	3 (75.0%)	
Madina	2 (14.3%)	12 (85.7%)	
Makkah	0 (0.0%)	5 (100.0%)	
Riyadh	6 (18.8%)	26 (81.3%)	
Tabuk	1 (5.9%)	16 (94.1%)	
Taif	4 (26.7%)	11 (73.3%)	

Participants' motives for conducting research

**Figure 1:**
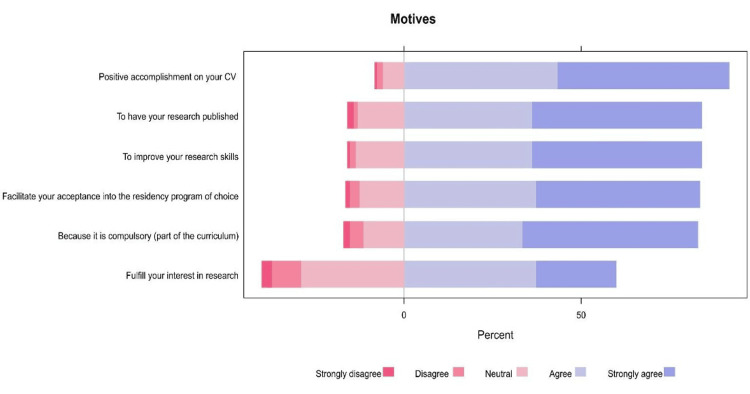
The proportions of participants' responses regarding the motives to conduct research.

A considerable proportion of residents agreed or strongly agreed that the primary motives for conducting research were to have positive accomplishments in their curriculum vitae (91.8%), improve their research skills (84.1%), and publish research (84.1%, Figure 1).

Participants' perceived barriers to conducting research

**Figure 2:**
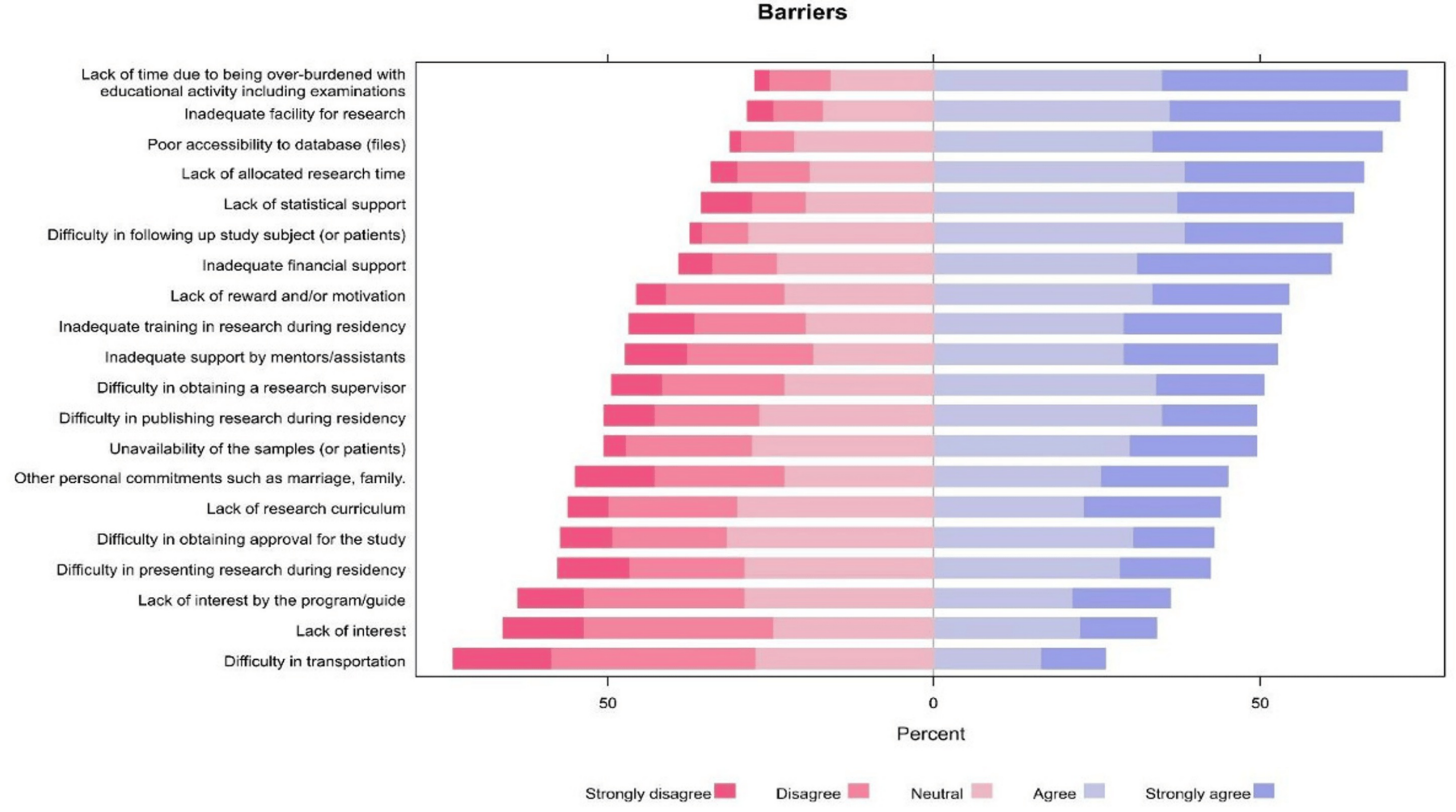
The proportions of participants' responses regarding the barriers to conduct research.

A significant proportion of residents agreed or strongly agreed that the main perceived barriers to conducting research included lack of time due to being overburdened with educational activities (72.6%), inadequate facilities for research (71.5%), and poor database accessibility (68.7%). Other significant barriers were the lack of allocated research time (66.0%), lack of statistical support (64.3%), and difficulty in following up with study subjects (62.7%, Figure 2). There was no significant association between the perceived barriers and residency levels.

## Discussion

Most residents (80.2%) published articles in journals, with significant differences according to age, year of residence, and program city. The overwhelming majority of residents were willing to participate in research workshops, with the main motivation being to have positive accomplishments in their CVs (91.8%). The main perceived barriers to research conduction were a lack of time (72.6%), inadequate facilities (71.5%), and poor database accessibility (68.7%).

During residency training, postgraduate doctors in preventive medicine learn how to develop and conduct research studies. This training is a crucial part of the curriculum and aims to develop highly skilled physician-scientists while boosting research productivity and quality [16]. This study is the first to examine attitudes, behaviors, and perceived barriers regarding research in preventive medicine residency programs [2]. Comprehending and addressing these perceptions can help enhance the research infrastructure and inspire physicians to become proficient clinical researchers [10,14,17-19].

This study showed that 96.2% of preventive medicine residents participated in research, with 80.2% having a publication. These rates are higher than those for most other specialties. This unusually high publication rate, compared to other specialties and settings, may reflect response bias, program-specific factors, or reporting differences, and should be interpreted with caution. Among family medicine residents, Al-Khaldi reported a 74% publication rate [3]. A study conducted by Soubhanneyaz et al. reported that 57% of family medicine doctors had no publications [12]. Moreover, AlEnazi et al. found that over 61% of residents of different medical specialties participated in research. However, only 18% published their work [2]. In India, only 15% of resident doctors had a previous publication [20].

The most common types of research projects in which residents participated were cross-sectional projects (80.8%), review articles (33.5%), and case reports (12.1%). The predominance of cross-sectional studies and limited engagement in more complex research designs may point to a gap in methodological training or mentorship, suggesting a need for broader research exposure during residency. The involvement of specific research types may vary by specialty. Family medicine doctors had a similar attitude to that of preventive medicine doctors, wherein the majority of published papers were cross-sectional (73-93%) and retrospective studies (7-10.7%) [2,3,12]. However, regardless of specialty, case reports were found to be the most common type of research among residents (34.5%) [14].

Despite the positive contribution of preventive medicine residents to research, only 36.8% had presented a research poster at a conference. The lack of resident participation in conferences and seminars is a significant issue that must be addressed. Residents should be motivated to present and share their studies at every opportunity. Furthermore, the majority of residents were motivated by CV improvement (91.8%), improved research skills (84.1%), and research publication (84.1%). This aligns with the work of Al-Khaldi, wherein 60% of the residents' contributions were for academic reasons [3].

Residents showed high motivation for research in this study, with 86.3% being ready to participate in research workshops, while 91.2% and 75.8% were willing to participate in community-related and clinical research, respectively. The same result was revealed by most surveys on this subject across different specialties [2,6-9,12,14]. In a study by Mitwalli et al., 93.7% of residents believed that research should be a mandatory part of the residency curriculum. Meanwhile, 92.2% agreed that training residents should participate in research [14]. Moreover, 70.7% of postgraduate doctors in India were willing to participate in workshops on research methodology [9]. Residents with the opportunity to publish during their residency may be inclined to pursue more publications in peer-reviewed journals. More research in this field will provide additional insights and enable the better allocation of resources in postgraduate training [5,21,22].

The research culture in Saudi Arabia has evolved significantly, reflecting changes in the educational and healthcare landscapes [14,15]. The country has strongly prioritized research, aiming to achieve global benchmarks in higher education according to the 2030 Vision [14]. Research productivity in the health sciences has increased notably, particularly between 2008 and 2017, indicating a strengthening of the research environment within healthcare [15]. However, the median worldwide ranking for Saudi Arabia remains low based on the total number of documents published [11]. Additionally, medical specialty research is gaining traction, focusing on improving healthcare standards and addressing local health issues [14]. Research among resident physicians reveals a growing awareness of the importance of research in medical training, although varying perceptions and attitudes may influence engagement levels [21]. Postgraduate medical research programs, along with research-focused staff, can contribute to establishing a sustainable research environment. Furthermore, faculty research productivity in public universities is framed as a crucial component of human capital investment, aligning with national goals to enhance educational outcomes [15,21]. International research partnerships and collaboration tend to enhance the productivity of researchers [21].

Despite the residents' involvement in research projects, they encountered some challenges. A lack of time due to being overburdened with educational activities was the primary barrier (72.6%), followed by inadequate facilities for research (71.5%) and a lack of statistical support (64.3%), which were the main perceived barriers around the globe and in various specialties [2-4,7,9,10,14,23-25]. The current preventive medicine program allows for dedicated research time; however, it may not be sufficient, possibly due to other necessary academic commitments [16]. It may be necessary to reassess the existing curriculum and increase the time allocated for research to improve the involvement of preventive medicine postgraduates in research.

Interestingly, certain obstacles that were particularly prevalent in this study did not appear to be the main obstacles in other studies [2,6,7,12]. These included poor database accessibility (68.7%) and difficulty in following up with study participants (62.7%). The unique research contexts and priorities of preventive medicine could amplify the challenges of database access and subject follow-up compared to more clinically focused specialties. Understanding these discipline-specific factors is essential to identify solutions to address these barriers.

Limitations

Despite our best efforts to ensure an adequate sample size for this study, it had some limitations. The study was based on convenience sampling; thus, it may not reflect the actual attitudes, behaviors, and perceived barriers of all postgraduate doctors. Furthermore, this study targeted only postgraduate Saudi residents in preventive medicine. To enhance the study's utility and impact, expanding the scope to include multiple medical specialties is essential, as it increases generalizability and enables meaningful comparisons across the healthcare system. Additionally, while the questionnaire was adapted from a previously validated tool, the full instrument was not included in this manuscript due to copyright constraints. This may limit the replicability of the study. Finally, there is potential for response bias owing to the study design. The reliance on self-reported data can introduce biases, such as social desirability bias, where participants may provide responses that they believe are more acceptable or favorable rather than their true behaviors. This can lead to inflated reports of knowledge or skills, ultimately affecting the validity of the findings. For example, while participants demonstrated a keen interest in participating in research activities and cited skill improvement as a motive, these responses may reflect the expectations and pressures they perceive from the program structure and academic environment.

## Conclusions

This study provides valuable insights into the research engagement of preventive medicine residents in Saudi Arabia, revealing a high publication rate among participants. The findings indicate significant demographic associations with publication rates, particularly among younger residents and those in their first year of residency. Despite these positive indicators, participants reported several barriers to conducting research, including a lack of time, inadequate facilities, and poor database accessibility. While causality cannot be inferred from this cross-sectional design, the reported barriers suggest potential areas for institutional improvement. A strategic approach to supporting research, such as offering technical assistance, improving infrastructure, and facilitating access to research tools, may help create a more enabling environment for residents. Additionally, initiatives like financial support and participation in academic conferences could encourage greater engagement. These strategies align with broader efforts to advance research capacity in line with Saudi Arabia's Vision 2030 goals.
